# Magnetic Purcell Enhancement by Plasmon-Induced Magnetic Anapole Mode in the Gap of Oblate Nano-Ellipsoid on Metal Mirror Structure

**DOI:** 10.3390/nano15181451

**Published:** 2025-09-20

**Authors:** Yafei Li, Jiani Li, Zhuangzhuang Xu, Xiufei Li, Songda Gu, Ze Li, Meng Wang

**Affiliations:** 1 Key Laboratory of Semiconductor Photovoltaic Technology and Energy Materials of Inner Mongolia Autonomous Region, School of Physical Science and Technology, Inner Mongolia University, Hohhot 010021, China; l1875204112@163.com (Y.L.); xiufeili@imu.edu.cn (X.L.); 0221122940@mail.imu.edu.cn (S.G.); 2 Research Center for Quantum Physics and Technologies, Inner Mongolia University, Hohhot 010021, China; jn-li@mail.imu.edu.cn (J.L.); xuzhuangzhuang@mail.imu.edu.cn (Z.X.)

**Keywords:** magnetic anapole mode, Purcell enhancement, nano-particle on mirror

## Abstract

Magnetic anapole states associated with the destructive interference between magnetic dipole and magnetic toroidal moments result in suppressed scattering accompanied by strongly enhanced near fields. Here, we demonstrate the existence of such modes in the gap of a gold oblate nano-ellipsoid on gold mirror (ONEOM) structures and observe a pronounced Purcell factor enhancement for magnetic dipole radiation upon introducing magnetic dipoles into the gap. We systematically investigate the dependence of the magnetic radiation Purcell factor on gap size and structural parameters. Notably, a 230-fold Purcell factor enhancement is achieved for the ONEOM configuration. This result highlights the potential of ONEOM structures in applications requiring efficient magnetic dipole emission, including nonlinear frequency conversion, plasmonic sensing, and single-photon sources.

## 1. Introduction

The Hamiltonian describing light–matter interactions during photoexcitation and relaxation processes is given by [1,2]:

(1) H=−p⋅E−m⋅B,

where **p** and **m** represent the electric and magnetic dipole moments, respectively.

Typically, magnetic dipole transitions exhibit orders-of-magnitude weaker amplitudes compared to electric dipole transitions [3,4]. The magnetic term dominates emission processes only when electric dipole transitions are forbidden.

However, with the advent of metamaterials and nanostructured systems, magnetic interactions have gained significance [2]. A comprehensive characterization of magnetic properties is essential for advancing both fundamental research and practical applications [5,6,7,8,9]. To investigate magnetic light–matter interactions, an effective magnetic “hot spot” resonator is required [10]. High-index dielectric nanostructures supporting Mie-type resonances have been extensively studied for magnetic field enhancement. However, the strongest magnetic fields remain confined within dielectric materials, inaccessible for direct interaction with matter [11,12,13]. Metallic nano-resonators can trap light at subwavelength scales via localized surface plasmon resonances (LSPRs), and plasmonic arrays are commonly used to create magnetic hotspots. Yet, conventional gap-mode magnetic dipole modes in multiple nanoparticles, which typically exhibit large mode volumes and insufficient magnetic field enhancement, are inadequate for studying light–matter interactions involving magnetic components [14,15,16,17].

To address the aforementioned limitations, we leveraged a classic nanoresonator architecture—the nanoparticle-on-mirror (NPOM) system—which allows for precise light manipulation at the single-emitter/molecule scale [18,19]. This configuration has been proven effective in tailoring optical interactions at the nanoscale, offering unique advantages for single-entity quantum optoelectronic studies [20,21]. Gaps with nanometer-scale separations between nanoparticles and substrates can be realized through molecular layer assembly [18,19] or atomic layer deposition [22,23]. These extreme electromagnetic field confinements enable applications such as strong light–matter coupling [24], plasmon-enhanced spectroscopy [8,25], and Purcell effects [20,21]. While NPOM has revolutionized the electric dipole decay rate enhancement in fluorescent emitters [26], magnetic Purcell enhancement via magnetic dipole transitions remains unexplored.

Similarly to electric-dipole Purcell effects [27,28,29], magnetic-dipole transitions can be controlled by the local density of optical states (LDOS) [30]. Although LDOS manipulation and magnetic dipole engineering have been explored in metallic mirrors and plasmonic arrays [5,20,31], low LDOS typically limits magnetic emitter enhancement. Therefore, achieving significant magnetic Purcell enhancement in NPOM requires inducing effective magnetic “hot spots” in the gap region [32]. Beyond radiating electric dipoles (ED) and subradiant magnetic dipoles (MD), anapole modes have garnered substantial theoretical and experimental attention over the past decade, owing to their non-radiating nature [32,33]. Encompassing both electric and magnetic variants, anapole modes enable the accumulation of near-field energy by suppressing radiative losses. Specifically, electric anapoles arise from the destructive interference between EDs and electric toroidal dipoles (ETD), which exhibit equal amplitude but opposite phase in the far field. Similarly, magnetic anapoles are induced by the coherent interplay between MDs and magnetic toroidal dipoles (MTD). The unique characteristics of anapole modes hold significant promise for applications such as field enhancement, metamaterials, and optical trapping. Recent theoretical investigations have demonstrated that magnetic “hotspots” can be excited within the magnetic anapole (MA) modes of NPOM structures [20,21,32,33], offering valuable prospects for exploring the magnetic Purcell effect.

In this work, we demonstrate the existence of MA states in a gold oblate nano-ellipsoid on gold mirror (ONEOM) system, arising from interference between MD and MTD. These MA states exhibit a strong magnetic field enhancement (∣H/H_0_∣ = 64) and extended spatial enhancement regions. By introducing virtual magnetic dipoles into the gap region, we theoretically investigate radiative magnetic Purcell enhancement under MA resonance conditions. Our analysis reveals that the Purcell factor exhibits dependence on gap size (2–10 nm) and structural parameters (diameter 100–200 nm). To our knowledge, a 230-fold Purcell enhancement in a metallic platform is predicted for a configuration with a 2 nm gap, 300 nm diameter, and 60 nm thickness, representing the strongest radiative magnetic Purcell factor reported for a plasmonic system within theoretical frameworks to date. We believe our research holds significant potential for advancing single-photon emission from magnetic dipole transition emitters (e.g., magnetic dipole transitions in rare-earth ions). Furthermore, this work may open up entirely new avenues for investigating magnetic Purcell enhancement within the nanoparticle-on-mirror NPOM structure.

## 2. Results and Discussion

**Figure 1:**
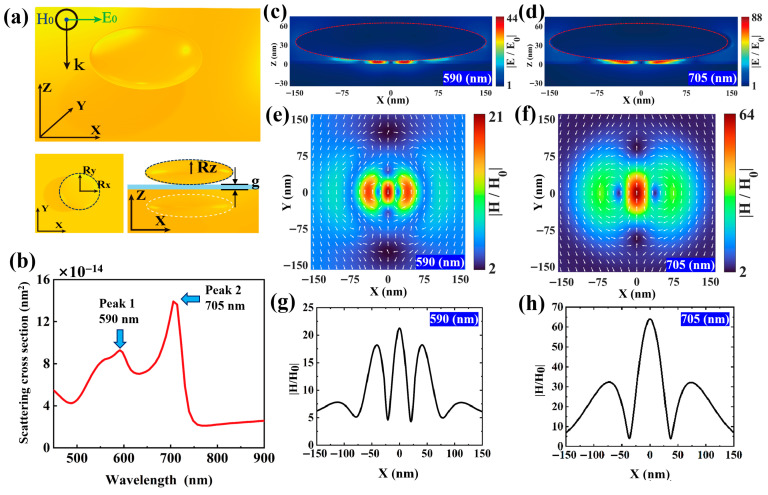
(**a**) Schematic diagram of the gold oblate nano-ellipsoid on gold mirror (ONEOM) system with coordinate system annotations. The incident plane wave propagates along the negative Z-axis, with polarization along the X-axis and magnetic field direction along the Y-axis. (**b**) Simulated far-field scattering spectra of the ONEOM structure with radii Rx = Ry = 150 nm, Rz = 30 nm, and a 5 nm gap. Two distinct scattering peaks are observed at 590 nm (Peak 1) and 705 nm (Peak 2). (**c**,**d**) Electric field distributions at the center of the ONEOM gap in the x-z plane at 590 nm and 705 nm, respectively. (**e**,**f**) Magnetic field distributions on the surface of the SiO_2_ isolation layer in the x-y plane at corresponding wavelengths. (**g**,**h**) The curve represents the magnetic field enhancement along the x-axis at y = 0 nm.

The schematic diagram of the ONEOM structure and corresponding coordinate system is presented in Figure 1a. The structure comprises a gold oblate nano-ellipsoid (with Rx = Ry > Rz) placed atop a gold mirror with a gap size of g. This configuration can be fabricated by drop-casting gold ellipsoidal nanoparticles synthesized via seed-mediated synthesis onto a gold substrate, where the 2 nm gap is achieved through molecular layer assembly. The far-field and near-field properties of this structure were investigated using a three-dimensional finite difference time domain (3D-FDTD) method (Details are provided in the Supporting Information S1). The gold permittivity was determined using experimental data reported in [34].

When Rx = Ry = 150 nm, Rz = 30 nm, and g = 5 nm, the far-field scattering spectra of NPOM exhibit peaks at 590 nm and 705 nm (Figure 1b), corresponding to second-order and first-order magnetic anapole (MA) states labeled as Peak 1 and Peak 2, respectively. To elucidate the MA resonance mechanism, we present near-field distributions including electric field maps in the x-z plane (Figure 1c,d), magnetic field H overlaid with magnetic flux lines in the x-y plane (Figure 1e,f) at wavelengths corresponding to these peaks and the curve represents the magnetic field enhancement along the x-axis at y = 0 nm (Figure 1g,h). The formation mechanism of the MA modes at 590 nm and 705 nm is illustrated in Figure S2, which shows a schematic of the magnetic anapole (MA) mode together with the supporting magnetic field intensity and electric field line distributions in the xz-plane.

At 590 nm (Peak 1), the electric field concentrates primarily at the gap edges of the magnetic region (Figure 1c). A localized electric dipole mode emerges in the bottom gold oblate nano-ellipsoid, generating a mirror image with antipodal phase distributions on the gold film surface. In addition, the near-field magnetic field displays four circular flux lines with opposite orientations within the gap region of the x-y plane (Figure 1e), and these flux lines generate two pairs of out-of-plane electric field spots. When combined with the poloidal magnetic field distribution, this observation indicates the simultaneous excitation of MD and MTD moments along the y-axis. The similar radiation patterns of the MD and MTD modes lead to destructive interference in the far field, thereby inducing a magnetic non-radiating resonance in the gap. Furthermore, four magnetic field nulls can be identified along the x-axis (Figure 1g), which further confirms the generation of a second-order magnetic anapole mode at this wavelength.

At 705 nm (Peak 2), the magnetic field lines form two counter-rotating loops on the central cross-section of the x-y plane (Figure 1f), which in turn generate two opposing out-of-plane electric field spots (Figure 1d). At this wavelength, energy is confined more strongly to the gap center—a characteristic that enables efficient light–matter interaction within the structure. Furthermore, two magnetic field nulls are identified along the x-axis, confirming the excitation of a first-order magnetic anapole mode (Figure 1h). By contrast, the field distribution at 590 nm is more complex, with significantly weaker magnetic field enhancement. Normalized magnetic field measurements indicate a peak enhancement of 21 × H_0_ at 590 nm, while the enhancement at 705 nm reaches 64 × H_0_. Notably, unlike electric anapoles that typically induce scattering valleys, magnetic anapoles exhibit minimal impact on the scattering spectrum due to the dominance of electric multipole radiation in plasmonic structures, explaining the absence of a valley in this case.

**Figure 2:**
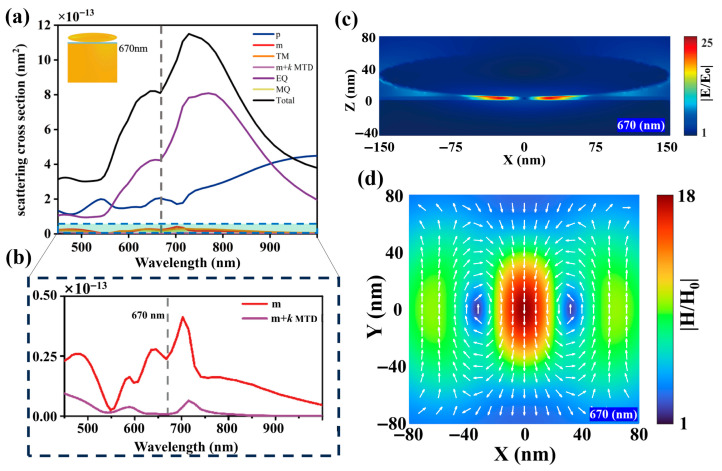
(**a**) Cartesian multipole decomposition of the sandwich structure composed of an Au oblate spheroid, a ${SiO}_{2}$ plate, and an Au plate. (**b**) Zoom-in view of the shaded region in (**a**). (**c**) Electric field distribution at the gap center in the x-z plane at a wavelength of 670 nm. (**d**) Magnetic field distribution on the surface of the silica spacer layer in the x-y plane at a wavelength of 670 nm.

To provide conclusive evidence of the magnetic anapole in ONEOM, a multipole decomposition of the scattering field or near-field is essential. While such decomposition is valid for isolated particles in homogeneous environments, the presence of a substrate—particularly one supporting surface wave excitation—complicates this analysis. We therefore consider a specialized sandwich structure comprising a Au oblate spheroid and a Au plate immersed in air, which closely resembles our target nanostructure (Rx = Ry = 150 nm, Rz = 30 nm, gap = 5 nm) while maintaining environmental homogeneity. We calculated the scattering contributions from the ED, MD, and electric quadrupole (EQ) moments, as well as the interaction term (m + k MTD) between MD and MTD, and the total scattering power of the sandwich structure. This involved expanding the oscillating charge-current distribution induced by the incident linearly polarized plane wave into a series of electric, magnetic, and toroidal multipole moments using FDTD-simulated total electric fields (Details are provided in the Supporting Information S2) [32]. The MA mode is characterized by the complete destructive interference between these moments, which mathematically manifests as $m_{car}\;$= −k${MTD}_{car}$. This condition effectively nullifies the net magnetic dipole contribution, as shown in Figure 2b, where the m + k MTD term vanishes at 670 nm, confirming destructive interference between MD and MTD. Furthermore, at this wavelength, the magnetic field lines in the x-y plane form two counter-rotating loops at the central cross-section (Figure 2d), which in turn generate two opposite out-of-plane electric field spots (Figure 2c). This distribution is highly consistent with the electromagnetic field distribution of the ONEOM structure at 705 nm, further confirming that an anapole mode is formed at 705 nm in the ONEOM structure. The small mismatch between the sum of multipole contributions and the total scattering cross-section in the sandwich structure is attributed to the fact that higher-order ETD moments were not included in our decomposition [32,35].

Within the multipole decomposition framework of the sandwich structure shown in Figure 2a, a distinct phenomenon emerges: concurrent destructive interference between the MD and MTD moments is accompanied by enhanced ED contribution and reduced total scattering intensity. In contrast, the ONEOM structure exhibits a marked deviation characterized by an anomalous scattering peak precisely at the 705 nm magnetic anapole wavelength. This behavior arises from the significantly stronger ED contribution in the ONEOM compared to its sandwich-structured counterpart, thereby altering the conventional balance of multipole interactions [32,33]. The present NPOM configuration could potentially enable new avenues for enhancing single-photon emission through magnetic dipole transitions.

The foundational proposal to modify spontaneous emission originated from Purcell’s 1946 work [36,37], where he demonstrated that coupling a quantum emitter to an electromagnetic resonator could enhance nuclear magnetic transition rates. This concept has since been extended to control electronic transitions in nanophotonic systems. To investigate the pronounced magnetic field enhancement in the MA state of the ONEOM structure arising from the interaction between light’s magnetic component and matter, we conducted a study of spontaneous emission from a magnetic emitter. This emitter features a specified normalized magnetic dipole moment intensity with polarization aligned along the axial y-axis orientation. Notably, the emitter exhibits no intrinsic loss mechanisms and was precisely positioned at the gap center where the magnetic field reaches its maximal magnitude.

**Figure 3:**
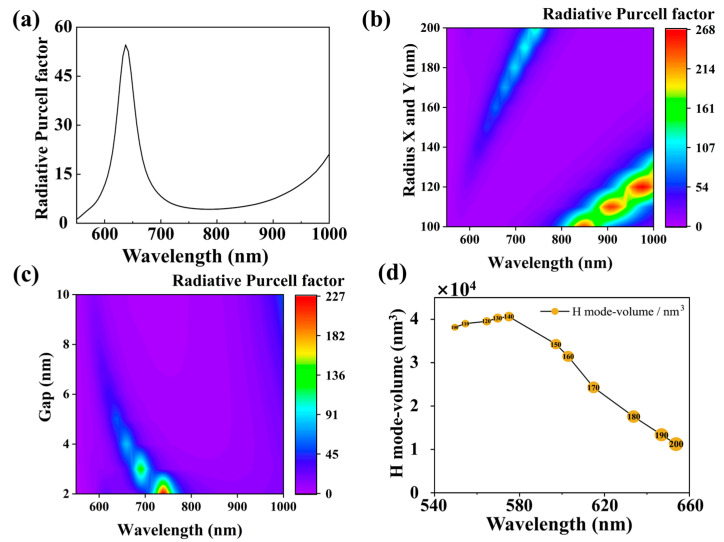
(**a**) Enhancement factor of far-field radiation decay rate ($F_{rad}$) for a magnetic dipole emitter located at the center gap of an ONEOM structure (with Rx = Ry = 150 nm, Rz = 30 nm, and gap thickness g = 5 nm). (**b**) Color map showing the dependence of the radiation magnetic Purcell factor on ellipsoid dimensions within the ONEOM structure. (**c**) Color map illustrating the relationship between the radiation magnetic Purcell factor and gap size in the ONEOM structure. (**d**) Magnetic mode volume distributions of elliptical nanospheres with diameters varying from 100 nm to 200 nm at the wavelength corresponding to the first magnetic anapole (MA) mode.

The far-field radiative decay rate enhancement factors $F_{rad}$ are plotted in Figure 3a. We observe significant enhancements reaching up to 55-fold at the wavelength corresponding to the first magnetic anapole (MA) mode.

To explore stronger Purcell effects, we investigated the influence of nano-ellipsoid size and gap dimensions on $F_{rad}$. Fixing Rz = 30 nm and g = 5 nm, we systematically enlarged Rx and Ry from 100 nm to 200 nm. This resulted in a redshifted peak for $F_{rad}$, as visualized in the contour map of Figure 3b. The map reveals two distinct branches corresponding to first-order and second-order MA modes. The maximum enhancement factor of 267-fold was achieved at λ = 980 nm for the first-order MA mode when Rx = Ry = 120 nm. Simultaneously, the second-order MA branch emerged and exhibited a continuous redshift with increasing dimensions, reaching 132-fold enhancement at λ = 744 nm for Rx = Ry = 200 nm. Next, we investigate the impact of the gap size between the gold oblate nano-ellipsoid and the gold mirror on the radiative Purcell factor. By fixing Rx = Ry = 150 nm and Rz = 30 nm, we observe a significant enhancement in $F_{rad}$, reaching a maximum value of 230-fold at g = 2 nm as shown in Figure 3c. It is important to note the specific limitations of the maps presented in Figure 3b,c. Due to the sparse, discrete sampling along the parameter axis (11 equidistant points for Figure 3b and 9 for Figure 3c), these particular maps are excellent for visualizing trends but are not suited for determining the PF value at an arbitrary parameter value. 

In this study, for an emitter in an electromagnetic environment, the modified far-field radiation rate is proportional to the local photon density of states (LDOS) and inversely proportional to the mode volume V: $\Gamma_{rad}/\Gamma_{0}\propto \rho /V$. The H-field mode volume V of the ONEOM structure is calculated as:

(2) $$V=\frac{\int H^{2}{dV}^{2}}{\int H^{4}dV}$$

The corresponding magnetic mode volume in the first magnetic anapole (MA) mode is demonstrated in Figure 3d. Moreover, as the diameter of the nano-ellipsoid increases, the mode volume associated with the magnetic dipole decreases, which correlates with the observed increase in the magnetic Purcell factor.

**Figure 4:**
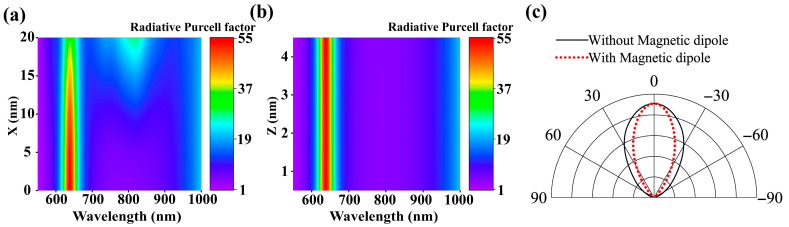
(**a**) Color map depicting the radiative magnetic Purcell factor of a magnetic-dipole emitter in the ONEOM structure with spatial variation along the x-axis (X). Here, X = 0 corresponds to the center of the gap region. (**b**) Color map illustrating how the radiative magnetic Purcell factor changes with gap distance for a magnetic-dipole emitter positioned at the center gap of the ONEOM system. (**c**) Normalized far-field radiation patterns in the XZ-plane at 705 nm (wavelength of the first magnetic anapole MA mode) with/without a magnetic dipole at the center gap of the ONEOM system.

We performed systematic investigations by fixing Rx = Ry = 150 nm, Rz = 30 nm and g = 5 nm. When the magnetic dipole was displaced along the x-axis at z = 2.5 nm (positions X = 0, 5, 10, 15, 20 nm), we observed significant enhancements in the effective radiative magnetic Purcell factor, as shown in Figure 4a. Specifically, a maximum Purcell enhancement of 55-fold was achieved at λ = 637 nm at the center (X = 0 nm), which gradually decreased to less than 35-fold when the emitter was displaced up to X = 20 nm.

In Figure 4b, the contour map demonstrates consistent Purcell enhancement (up to 55-fold) across varying emitter positions along the z-axis at the gap center (X = Y = 0 nm). This observation suggests universal magnetic field confinement within the nanogap region between the oblate nano-ellipsoid and the gold film. Such spatial insensitivity of the Purcell effect implies practical advantages for device fabrication, as molecular positioning within the gap does not require nanometric precision.

In Figure 4c, the black line represents the far-field radiation pattern of the bare ONEOM system at the wavelength of the first magnetic anapole (MA) mode, with its main lobe confined within the angular range of −30° to 30° along the Z-direction. The red line corresponds to the emission pattern of the magnetic dipole emitter associated with the emission peak in Figure 3a. Notably, the radiation lobes (black and red lines) nearly overlap completely, indicating that the emission from the magnetic dipole within the nanoscale plasma gap is dominated by the MA mode, while contributions from other modes can be neglected.

## 3. Conclusions

In this study, we demonstrate that magnetic anapole (MA) states can be excited in the gap of an ONEOM system through the interference between magnetic dipole and toroidal moments, which share identical far-field radiation patterns. By constructing a plasmonic structure comprising a gold oblate nano-ellipsoid and a gold mirror, we achieve a pronounced magnetic field enhancement (|H/H_0_| = 64) with a uniform spatial distribution within the nanogap region. This configuration not only enables efficient assembly of magnetic dipole-emitting materials but also establishes a strong MA resonance condition.

When a magnetic dipole (MD) emitter is introduced into the gap, we observe that the radiative decay rate can be enhanced by up to 230-fold under the condition of optimized ideal system parameters (Rx = 150 nm, Ry = 150 nm, Rz = 30 nm, g = 2 nm), with strong coupling to the second-order magnetic plasmon mode (magnetic anapole mode). Furthermore, we systematically investigate the Purcell enhancement mechanism by varying the emitter position and gap geometry. The results reveal that the magnetic Purcell factor exhibits significant dependence on both gap size (g) and nano-ellipsoid dimensions (Rx, Ry, Rz). Notably, a record Purcell enhancement of 267-fold is achieved, surpassing all previously reported values for magnetic dipole emitters in plasmonic cavities.

## Data Availability

The original contributions presented in this study are included in the article/Supplementary Material. Further inquiries can be directed to the corresponding authors.

## Supplementary Materials

The following supporting information can be downloaded at: https://www.mdpi.com/article/10.3390/nano15181451/s1. S1: Numerical simulations; S2: Multipole decomposition; S3: Radiative magnetic Purcell effect; S4: Schematic of the magnetic anapole (MA) mode formation; S5: Radiative magnetic Purcell factor under different polarizations.
