# Supplementary material for: Magnetic Purcell Enhancement by Plasmon-Induced Magnetic Anapole Mode in the Gap of Oblate Nano-Ellipsoid on Metal Mirror Structure
Source: Nanomaterials (Basel). 2025 Sep 20;15(18):1451. doi: 10.3390/nano15181451 (PMC12472662; doi:10.3390/nano15181451)
Supplement: Supplementary file 1 [file nanomaterials-15-01451-s001.zip › nanomaterials-3752748-supplementary.pdf]

Supporting information for "Magnetic Purcell Enhancement by Plasmon-Induced Magnetic Anapole Mode in the Gap of Oblate Nano-Ellipsoid on Metal Mirror Structure"

Yafei Li [1,‡], Jiani Li [2,‡], Zhuangzhuang Xu [2,‡], Xiu-Fei Li [1], Songda Gu [1], Ze Li [1,2,*], and Meng Wang [1,2,*]

[1] Key Laboratory of Semiconductor Photovoltaic Technology and Energy Materials of Inner Mongolia Autonomous Region, School of Physical Science and Technology, Inner Mongolia University, Hohhot,Inner Mongolia 010021, China.

[2] Research Center for Quantum Physics and Technologies, Inner Mongolia University, Hohhot, Inner Mongolia 010021, China.

‡ These authors contributed equally to this work.

* Correspondence: Corresponding author: li@imu.edu.cn; wangmeng@imu.edu.cn

S1: Numerical simulations

We employed LUMERICAL FDTD (finite difference time domain) SIMULATION to characterize the far-field and near-field optical magnetic responses of the gold-oblate nano-ellipsoid on gold mirror (ONEOM) structure. In these simulations, a linearly polarized light source was configured as a total-field/scattered-field (TFSF) excitation, impinging normally onto the ONEOM structure. To precisely quantify the total scattering cross section, six 2D discrete Fourier transform (DFT) monitors were positioned within the scattered field region. Perfectly matched layer (PML) boundary conditions were implemented to eliminate artificial reflections. The simulation duration was set to 1000 fs to ensure numerical convergence. For the multipole decomposition of the sandwich structure, a 3D DFT monitor was employed, coupled with a 3D index monitor of matching dimensions to capture the electric field distribution.

To enhance computational efficiency, a non-uniform meshing strategy was implemented based on convergence tests with varying mesh sizes in the gap region (Fig. S1). These tests confirmed that the selected mesh sizes have a negligible influence on the simulation results. Accordingly, Yee cells of size 1 nm × 1 nm × 1 nm were used in the narrow gap region, while larger cells of 2 nm × 2 nm × 2 nm were adopted for other regions.

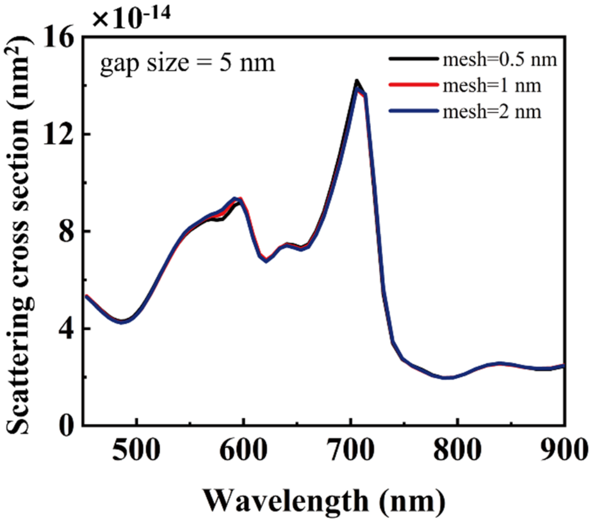

Figure S1. Convergence tests for varying mesh sizes in the gap region, while maintaining a constant gap size of 5 nm.

S2: Multipole decomposition

The different moments in the multipole decomposition were computed from the calculated distribution of the light-induced polarization, $\mathbf{P(r)} = \boldsymbol{\varepsilon_0}(\boldsymbol{\varepsilon_s}\mathbf{(r)} - \mathbf{1})\mathbf{E(r)}$, where ε_0 is the permittivity of vacuum, ε_r is the relative permittivity of the nano-ellipsoid and E(r) is the electric field induced by the incident light wave. The gold structure as nonmagnetic mediums, the multipole moment contributions to the far-field radiation including the electric dipole ED, magnetic dipole MD, electric quadrupole EQ, magnetic toroidal dipole MTD and magnetic quadrupole MQ, can be calculated as:

$$\mathbf{ED}: \mathbf{p}_{\text{car}} = \int \mathbf{P}(\mathbf{r}) d^3\mathbf{r}, \tag{1}$$

$$\mathbf{MD}: \mathbf{m}_{\text{car}} = -\frac{i\omega}{2} \int [\mathbf{r} \times \mathbf{P}(\mathbf{r})] d^3\mathbf{r}, \tag{2}$$

$$\mathbf{EQ}: \mathbf{Q}_{\alpha\beta}^{car} = 3\int \left\{ \mathrm{r}_\alpha \mathrm{P}_\beta(\mathbf{r}) + \mathrm{r}_\beta \mathrm{P}_\alpha(\mathbf{r}) - \frac{2}{3}\big(\mathbf{r} \cdot \mathbf{P}(\mathbf{r})\big)\delta_{\alpha\beta} \right\} d^3\mathbf{r}, \tag{3}$$

$$\mathbf{MTD}: \mathbf{T}_{\text{car}}^{(mag)} = i\omega \frac{k_0^2}{20} \int \mathrm{r}^2 [\mathbf{r} \times \mathbf{P}(\mathbf{r})] d^3\mathbf{r}, \tag{4}$$

$$\mathbf{MQ}: \mathbf{M}_{\alpha\beta}^{car} = \frac{\omega}{3i} \int \{ [\mathbf{r} \times \mathbf{P}(\mathbf{r})]_\alpha \mathrm{r}_\beta + [\mathbf{r} \times \mathbf{P}(\mathbf{r})]_\beta \mathrm{r}_\alpha \} d^3\mathbf{r}, \tag{5}$$

where $\mathbf{p}_{\text{car}}$,$\mathbf{m}_{\text{car}}$,$\mathbf{Q}_{\alpha\beta}^{car}$,$\mathbf{T}_{\text{car}}^{(mag)}$, $\mathbf{M}_{\alpha\beta}^{car}$ are the Cartesian electric dipole, magnetic dipole, electric quadrupole, magnetic toroidal dipole and magnetic quadrupole moments respectively, with α, β, γ = x, y, z. ω and k_0 are the angular frequency and wavenumber of the incident light and r is the position vector. The total scattering cross section aggregates contributions from all multipoles:

$$\boldsymbol{\sigma}_{\textbf{scat}} \approx \frac{k_0^4}{6\pi\varepsilon_0^2|\mathbf{E}_{\text{inc}}|^2} \Big(|\mathbf{p}_{\text{car}}|^2 + \mu_0 |\mathbf{m}_{\text{car}} + \mathbf{T}_{\text{car}}^{(mag)}|^2 + \frac{k_0^2}{120} \sum |\mathbf{Q}_{\alpha\beta}^{car}|^2 + \frac{3k_0^2\mu_0}{40} \sum |\mathbf{M}_{\alpha\beta}^{car}|^2 \Big) \tag{6}$$

The second term in the expansion explicitly contains the interaction between MD and MTD moments, denoted as MD+MTD.

S3: Radiative magnetic Purcell effect

For emitters interacting with structured environments rather than free space, the modified emission rate is governed by Fermi's golden rule:

$$\Gamma_g = \frac{2\pi}{\hbar^2}\sum_f |\langle f|H_I|i\rangle|^2\,\delta(\omega_i - \omega_f), \tag{7}$$

where $|i\rangle$ and $|f\rangle$ denote the initial and final states. The interaction Hamiltonian H_I as shown in Equation 1. In this study, we focus on magnetic dipole emission, allowing us to neglect the electric contribution. This simplifies the Purcell factor to:

$$\Gamma_g = \frac{\pi\omega}{3\hbar}\left(\mu_0|\mathbf{m}|^2\rho_B(r,\omega)\right), \tag{8}$$

where $\rho_B(r,\omega)$ is the magnetic local density of states (MLDOS), proportional to the magnetic field intensity at the emitter location.

The Purcell factor inherently accounts for both radiative and nonradiative decay channels. In this study, we focus exclusively on the radiative Purcell effect, which quantifies the enhancement of the radiative emission rate Γ_{rad} relative to the free-space rate Γ_0. Following the established formalism, this radiative Purcell factor F_rad is defined as:

$$F_{rad} = \frac{\Gamma_{rad}}{\Gamma_0} = \frac{P_{rad}}{P_0}, \tag{9}$$

where P_{rad} and P_0 represent the far-field radiation powers in the presence and absence of the optical nanostructures, respectively. This relationship can also be expressed as:

$$F_{rad} = \frac{P_{tot}}{P_0} - \frac{P_{abs}}{P_0}, \tag{10}$$

with P_{tot} denoting the total emission power and P_{abs} the power absorbed by the cavity.

In FDTD simulations, a localized magnetic emitter can be modeled as an oscillating current source with the radiation power given by:

$$P_0 = \frac{\omega^4|\mu|^2}{12\pi\varepsilon_0 c^3}, \tag{11}$$

To compute the far-field radiation power (Prad), six surrounding frequency-domain (FD) power monitors were positioned around the entire system (nanoparticle and emitter). The radiated power is determined by integrating the Poynting vector S over the closed monitoring surface, defined by these monitors:

$$P_{rad} = \int_{\Sigma} \boldsymbol{s}\cdot\boldsymbol{n}\,ds, \tag{12}$$

where n is the unit normal vector to the surface Σ.

S4: Schematic of the magnetic anapole (MA) mode formation

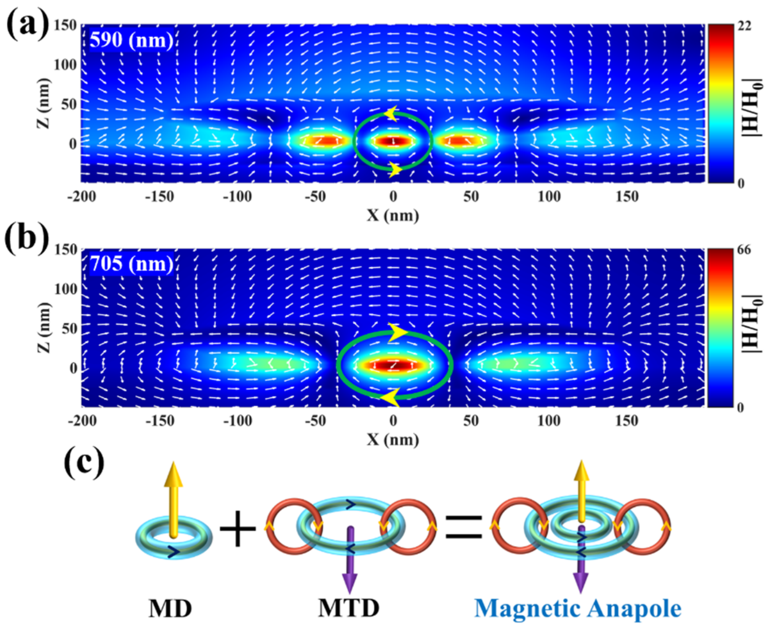

Figure S2. Magnetic field intensity and electric field lines in the xz-plane at (a)590 nm and (b)705 nm. (c)Schematic of the magnetic anapole (MA) mode formation.

To provide clearer evidence for the formation of the magnetic anapole (MA) modes at 590 nm and 705 nm, we have supplemented the magnetic field intensity distributions and electric field lines in the xz-plane. As illustrated in the main text's Figs. 1e and 1f, the counter-circulating magnetic vortices in the xy-plane induce out-of-phase electric dipoles (EDs), as shown in Figs. 1c and 1d. These out-of-phase EDs, in turn, generate a circulating electric field that creates a MTD at the gap center. This MTD oscillates out-of-phase with the MD shown in Figure S2, ultimately leading to the formation of the MA mode.

S5: Radiative magnetic Purcell factor under different polarizations

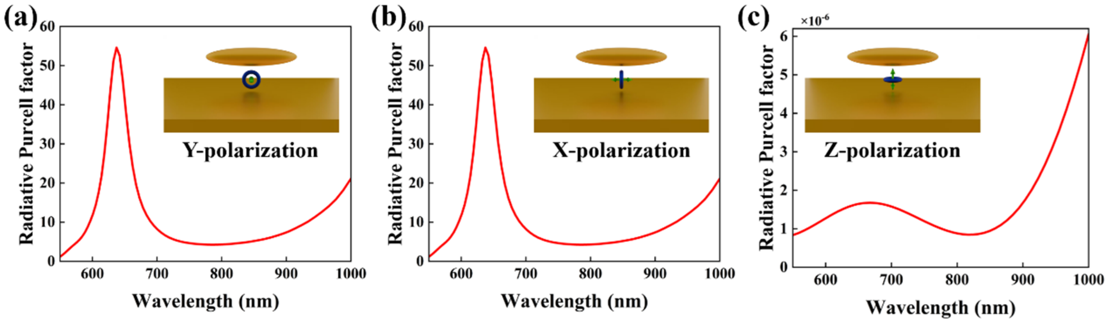

Figure S3. Radiative magnetic Purcell factor at gap center with magnetic dipole polarized along the (a) y-axis, (b) x-axis and (c) z-axis.

We calculated the Purcell enhancement factor (PF) of the magnetic dipole emitter at different orientations and found that dipoles oriented in the x-y plane can achieve effective Purcell enhancement. In contrast, z-polarized dipoles cannot excite the magnetic anapole mode in the NPOM structure, leading to a negligible radiative magnetic Purcell factor.
